# Diversity of culturable bacterial isolates and their potential as antimicrobial against human pathogens from Afar region, Ethiopia

**DOI:** 10.1128/spectrum.01810-24

**Published:** 2024-10-04

**Authors:** Sisay Demisie, Dong-Chan Oh, Dawit Wolday, Tobias F. Rinke de Wit, Adugna Abera, Geremew Tasew, Abebe Mekuria Shenkutie, Sisay Girma, Ketema Tafess

**Affiliations:** 1 Department of Applied Biology, School of Applied Natural Science, Adama Science and Technology University, Adama, Ethiopia; 2 Natural Products Research Institute, College of Pharmacy, Seoul National University, Seoul, South Korea; 3 Depatment of Biochemistry and Biomedical Sciences, McMaster University, Hamilton, Canada; 4 Department of Global Health, Amsterdam Institute for Global Health and Development (AIGHD), Amsterdam University Medical Centre, University of Amsterdam, Amsterdam, the Netherlands; 5 Ethiopian Public Health Institute, Addis Ababa, Ethiopia; 6 Department of Health Technology and Informatics, The Hong Kong Polytechnic University, Hong Kong, China; 7 College of Veterinary Medicine and Agriculture, Addis Ababa University, Bishoftu, Ethiopia; 8 Institute for Microbiology, University of Veterinary Medicine Hannover, Hannover, Germany; 9 Institute of Pharmaceutical Sciences, Adama Science and Technology University, Adama, Ethiopia; Institute of Microbiology, Chinese Academy of Sciences, Beijing, China

**Keywords:** secondary metabolites, antimicrobial activity, MALDI-TOF MS, WGS, GC–MS

## Abstract

Antimicrobial resistance is a growing global concern exacerbated by the scarcity of new medications and resistance to current antibiotics. Microbes from unexplored habitats are promising sources of natural products to combat this challenge. This study aimed to isolate bacteria producing secondary metabolites and assess their antimicrobial efficacy against human pathogens. Soil and liquid samples were collected from Afar region, Ethiopia. Bacterial isolates were obtained using standard serial dilution techniques. Antimicrobial activity was evaluated using agar plug and well diffusion methods. matrix-assisted laser desorption/ionization time-of-flight-mass spectrometry (MALDI-TOF MS) and whole-genome sequencing (WGS) were conducted for the isolate exhibiting the highest antimicrobial activity. Secondary metabolites were extracted and analyzed using gas chromatography-mass spectra (GC-MS). In this study, 301 bacteria isolates were identified, of which 68 (22.6%) demonstrated antagonistic activity against at least one reference pathogen. Whole-genome sequencing revealed that Sl00103 belongs to the genus Bacillus, designated as Bacillus sp. Sl00103. The extract of Sl00103 showed zones of inhibition ranging between 17.17 ± 0.43 and 26.2 ± 0.4 mm against bacterial pathogens and 19.5 ± 0.44 to 21.0 ± 1.01 mm against *Candida albicans*. GC-MS analysis of ethyl acetate and *n*-hexane extracts identified major compounds including (R,R)-butane-2,3-diol; 3-isobutylhexahydropyrrolo[1,2a] pyrazine-1,4-dione; cyclo(L-prolyl-L-valine); and tetradecanoic acid, 12-methyl-, methyl ester; hexadecanoic acid, methyl ester among other. In conclusion, this study isolated several promising bacterial strains from the Afar region in Ethiopia, with strain Sl00103 (Bacillus sp. Sl00103) demonstrating notable antimicrobial and antioxidant activities and warranting further studies.

**IMPORTANCE:**

Antimicrobial resistance (AMR) is an escalating global health threat affecting humans, animals, and the environment, underscoring the urgent need for alternative pathogen control methods. Natural products, particularly secondary metabolites from bacteria, continue to be a vital source of antibiotics. However, microbial habitats and metabolites in Africa remain largely unexplored. In this study, we isolated and screened bacteria from Ethiopia’s Afar region, characterized by extreme conditions like high temperatures, volcanic activity, high salinity, and hot springs to identify potential bioactive compounds. We discovered diverse bacterial isolates with antimicrobial activity against various pathogens, including strain Sl00103 (Bacillus sp. Sl00103), which demonstrated significant antimicrobial and antioxidant activities. GC-MS analysis identified several antimicrobial compounds, highlighting strain Sl00103 as a promising source of secondary metabolites with potential pharmaceutical applications and warranting further investigation.

## INTRODUCTION

Antimicrobial resistance (AMR) presents a significant and increasingly alarming global challenge to the health of humans, animals, and the environment. This is attributed to the rise, dissemination, and endurance of bacteria that are resistant to multiple drugs, commonly referred to as “superbugs” (1, 2). Multidrug-resistant (MDR) bacteria develop within humans, (domestic) animals, and in the environment, creating ample opportunities for exchange of genetic material (2–4). The rapid rise in antibiotic resistance (AR) and the paucity of novel antimicrobial medicines has ever increasing global attention as a serious problem (5–7). The increase in antibiotic-resistant pathogens implies a decrease in the accessibility of current antimicrobial agents to address them (8). The estimate is that by 2050, the currently existing antibiotics will no longer be effective to treat human and animal infectious diseases. This raises the need to search for alternative methods of controlling infectious pathogens in the future.

Natural products, including secondary metabolites from bacteria, remain a major source of global antibiotics. Most of the antibiotics used to treat infectious diseases were discovered during the 1950s−1970s, a period often referred to as the “golden era” of antibiotic discovery (9). However, in the past three decades, there has been a significant decline in the discovery of new antibiotics from natural sources, particularly bacteria (10). This decline is largely due to the high rate of rediscovery of known molecules with existing resistance mechanisms and the high proportion of hits with significant cytotoxicity or poor Absorption, Distribution, Metabolism, Excretion, and Toxicity (ADMET) properties (11). To overcome these limitations and uncover new antibacterial agents, it is essential to move beyond commonly studied groups of secondary metabolite producers, such as actinomycetes, and explore the vast, largely uncharted microbial diversity found in extreme environments like volcanic areas, deserts, deep-sea habitats, and high-salinity regions (12). Many of these organisms have adapted physiologies that enable them to produce unique bioactive metabolites, including antibiotics, compared to mesophilic counterparts.

Expanding the exploration of these untapped biological resources is crucial for future therapeutic development in both academic and industrial settings. In this regards, Ethiopia’s rich biodiversity and diverse climatic zones (13) present opportunities for discovering novel antibiotic-producing strains. Up to today, the microbial habitats and metabolites in this region of Africa remain underexplored (14). The objective of this study is to isolate and screen bacteria from Ethiopia’s Afar region—characterized by extreme conditions such as the desert ecosystems, volcanic activity, high salinity, and hot springs for their potential to produce bioactive compounds.

## MATERIALS AND METHODS

### Sample collection and processing

Eighteen samples were collected from diverse locations within the Dallol Depression and other areas in Afar region, Ethiopia (Table 1; Fig. S1). Sample collection was performed as previously outlined by Prashanthi et al. (15). Briefly, each sample comprised 250 g of soil collected at a depth of 5–10 cm in sterile bags, along with 100 mL of brine collected in plastic tubes. Subsequently, all samples were transported to the Laboratory of the Institute of Pharmaceutical Science at Adama Science and Technology University for detailed analysis.

**TABLE 1:** The geo-coordinates of sample collection sites

S.no	Sample site	Sample		Coordinate locations		
		Site code	Sample	Latitude	Longitude	Altitude
1	Sulfur lake	Sl001	Soil	14.214281	40.309214	−92
		Sl002	Soil	14.237583	40.297803	−89
		Sl003	Soil	14.237462	40.298951	−89
		Sl004	Soil	14.237582	40.297803	−93
		Sl005	Soil	14.237402	40.298512	−100
		Sl006	Soil	14.234379	40.301758	−102
		Sl007	Soil	13.945062	40.372872	−125
		Sl008	Liquid	14.237591	40.298274	−90
2	Asahil Lake	Asl011	Liquid	14.115998	40.348176	−132
3	Alalobad	Al001	Soil	11.622245	41.013788	400
		Al002	Soil	11.642075	41.014575	400
		Al003	Soil	11.622075	41.813788	400
		Al004	Soil	11.642075	41.014575	400
		Al005	Liquid	11.622275	41.813788	88
4	Wosema Kebele	Ws001	Soil	13.288191	39.876413	1,088
5	Amedela	Am001	Soil	14.115998	40.348176	−124
6	Lake Afrera	Af001	Soil	13.220131	40.874831	−110
7	Lake Afrera	Af002	Liquid	13.220131	40.874831	−110

### Bacterial isolation

Soil samples were grounded, sieved (250 µm), air-dried overnight, and sterilized at 60°C for 30 min. Bacterial isolation followed a previously described method with minor modifications (16). Briefly, 1 g of soil was subjected to serial dilution ranging from 10^−1^ to 10^−7^. From the 10^−3^ and 10^−5^ dilutions, 100 µL was inoculated onto nutrient agar plates containing nystatin and cycloheximide (40 and 50 µg/mL, Sigma-Aldrich, Steinheim, Germany) to inhibit fast bacterial and fungal overgrowth. For the liquid sample, 100 µL was directly inoculated. Plates were then incubated at 28°C for 24–72 h. Suspected colonies were isolated onto separate nutrient agar plates to obtain pure cultures. Pure cultures were preserved in sterile vials with 20% glycerol at −20°C for subsequent analysis.

### Screening of bioactive isolates

Test pathogens were transferred to sterile phosphate-buffered saline (PBS) to match turbidity equivalent to 0.5 McFarland standards, corresponding to a cell density of 10^6^–10^8^ CFU/mL for bacteria and 2.5 × 10^3^ CFU/mL for fungi. Optical densities of the suspensions were matched to 0.5 McFarland standards at 530 nm using a UV-visible Spectrophotometer (OPTIZEN 2120UV, Moscow). Active isolates were initially screened against seven human bacterial pathogens (*E. coli* ATCC 25922, *S. aureus* ATCC 25923, *E. faecalis* ATCC 29212, *P. aeruginosa* ATCC 27853, *A. baumannii* ATCC 19606, *S. typhi* ATCC 26531, *S. pyogenes* ATCC 12204) and one pathogenic fungus (*C. albicans* ATCC 10231) using the agar plug diffusion method (17). Inhibition zones were observed and recorded in millimeters. Sixteen (16) bioactive isolates that showed high inhibition zones in the primary screening were selected for biochemical and MALDI-TOF characterization and secondary antimicrobial activity screening using the well diffusion method as previous described (18).

### Biochemical and MALDI-TOF MS

Bacterial isolates grown overnight in Luria Bertani Broth (LB broth) were used for Gram staining. Colony morphology of the isolates was characterized Bergey’s manual for the identification of Bacillus species (19). For biochemical test, a loopful of overnight culture was inoculated into LB broth and cultured for an additional 24 h at 30°C. Then, culture suspension was utilized for biochemical tests following standard protocols described by Masi et al. (20). For MALDI-TOF MS test, isolates were also screened for enzyme production using carboxymethylcellulose, starch, gelatin, and casein. Single colonies were used for mass spectra analysis in a MALDI Biotyper Microflex LT (Bruker Daltonics, Bremen, Germany). Proteomic spectra generated by MALDI-TOF MS were compared against the reference spectra using Bruker MALDI-TOF Biotyper software to obtain identification with a confidence score (21).

### Growth optimization for Sl00103

Optimum growth conditions was determined with respect to the carbon sources (2%, wt/vol, each of glucose, fructose, mannitol, and sucrose) and nitrogen sources [0.6% wt/vol each of yeast extract, peptone, (NH4)2SO4, and NaNO3], as well as incubation temperatures (25°C, 30°C, 35°C, and 40°C), pH levels using sodium phosphate (pH 6.5), potassium phosphate (pH 7–7.5) , tris-HCl (pH 8), and NaCl concentrations (0.0025 g/mL, 0.005 g/mL, 0.01 g/mL, and 0.02 g/mL). The basal growth medium [(g/L) consisting of KH2PO4 0.5 g, K2HPO4 0.5 g, CaCl2 0.1 g, NaCl 0.2 g, MgSO4.7H2O 0.5 g, MnSO4.7H2O 0.01 g, FeSO4.7H2O 0.01 g, and NH4NO3 1.0 g] was used. Cultures were incubated for 96 h at 150 rpm. Optical density was measured in triplicate, and the data were recorded (22).

### Extraction of secondary metabolites from Sl00103

Extraction was conducted using microbial fermentation process as previously described (14, 23). Fresh inoculum incubated for 48 h at 30°C was transferred into 500 mL of LB broth and malt extract media in 1,000 mL Erlenmeyer flasks. The inoculum was then incubated at 30°C and 160 rpm with continuous shaking until the growth reached stationary phase. After fermentation, the supernatant was collected by centrifugation at 10,000 rpm for 5 min. The supernatant containing the bioactive metabolite was further filtered using Whatman No. 1 filter paper. The supernatant was collected and mixed with an equal ratio of ethyl acetate and *n*-hexane. The bioactive compound-containing organic solvent phase was then separated from the aqueous phase in a separatory funnel, collected, and evaporated in a vacuum rotary evaporator at 90 rpm and 45°C. The completely dried residues from each isolate were weighted separately using a balance and dissolved in dimethyl sulfoxide (DMSO) and placed in small vials at 4°C to determine the antimicrobial activity and further analysis.

### Antimicrobial activities of the extracts of Sl00103

The ethyl acetate and *n*-hexane extracts of the bioactive isolate were tested via the well diffusion method (17). Test pathogens were swabbed onto Muller Hinton Agar plates, and using a sterile cork-borer, the wells (6 mm) were created in the agar plate. Then, the wells loaded were with 100 µL of crude extract. Positive controls included Ciprofloxacin (10 mg/mL) and Amphotericin B liposome (8 mg/mL), while 10% DMSO served as the negative control. Inhibition zones were measured and recorded after 24 h of incubation. The experiment was conducted in triplicate.

### Evaluation of MIC, MBC, MFC, and MBIC extracts of Sl00103

A 10 mg/mL stock solution of the ethyl acetate extract of secondary metabolites was used for determining the values of MIC, MBC, MFC, and MBIC. Then, the growth assay was conducted using the broth micro dilution method in a 96-well microtiter plate. A total volume of 50 µL of sample stock solution was diluted twofold to give final concentration of 500, 250, 125, 62.5, and 31.25 µg/mL. Inoculum was prepared with the standardized of McFarland of 1.5 × 10^8^ CFU/mL for bacteria and 0.5 mL of 10^5^ for *C. albicans*. Then, 10 µL inoculum was added to each well and grown for 24 h at 37°C for bacteria and 48 h at 30°C for *C. albicans*. The concentration at which the extract exhibited no visible growth compared to the negative control was designated as the MIC value. To ensure the quality, the study involved growth control (pathogens with antimicrobial agents) and sterility control (media without pathogens). Finally, cultures with no visible growth were then transferred onto appropriate media. The MBCs and MFCs were calculated as the concentration that prevented growth of more than 99.9% of microorganisms after incubation for 24 h at 28 ^o^C or 37°C.

### Determinations of antioxidant activities

The antioxidant activity was conducted by using the DPPH free-radical scavenging assay (24). A 0.1 mM DPPH solution was prepared in methanol and kept in the dark for 30 min to complete the reaction. The ethyl acetate dilutions were prepared at 500, 250, 125, and 31.25 µg/mL. The same concentrations of ascorbic acid were used as a standard, and the sample-free DPPH solution was used as a negative control. After mixing 1 mL of DPPH solution with 3 mL of prepared samples, the mixture was incubated at room temperature in a dark place for 30 min, and the absorbance was measured at 517 nm. The percentage of radical scavenging assay (RSA) was calculated using the following formula: Percentage of RSA = [(*A* – *B*)/*A*] × 100, where *A* is absorbance of DPPH control and *B* is absorbance of DPPH in the presence of extract/standard. The percentage of RSA was calculated for both metabolite and standard.

### Gas chromatography-mass spectra analysis

GC-MS analysis of bacterial metabolite was performed by a GC (7890B, Agilent Technologies) coupled with an MS (5977A Network, Agilent Technologies). Helium was used as a carrier gas with a 4 min solvent delay and a split less injection/purge time of 1.0 min with diﬀerent ﬂow rates and runtime. In the ethyl acetate extract, the temperature increase was 160–280°C, the ﬂow rate was 1.2 mL/min, and the runtime was 30 min. For *n*-hexane, the temperature was increased 160–300°C, the ﬂow rate was 1 mL/min, and the runtime was 32 min. Mass spectra were recorded in an electron-impact mode, with ionization energy of mode at 70 eV, scanning the 33–550 *m*/*z* range. The secondary metabolite produced by the bioactive compounds produce bacteria were identified by comparing the mass spectra of the compounds in oils with those in the database of the NIST11 GC-MS libraries (25).

### Whole-genome sequencing (nanopore MinION sequencing) of Sl00103

Genomic DNA from Sl00103 was extracted using the QIAamp BiOstic Bacteremia DNA Kit (Qiagen, Germany). The concentration and purity of the DNA were assessed using NanoDrop and Qubit technologies. Subsequently, the DNA was sent to Hong Kong Polytechnic University for library preparation and sequencing. The library preparation involved barcoding 50 ng of genomic DNA, which was pooled with 11 other samples, followed by ligation of nanopore adapters. The sequencing was performed on a MinION Mk1C machine according to the manufacturer’s instructions.

### Strain identification

Genome *de novo* assembly was performed with Flye version v2.9.1-b1780 Flye version v2.9.1-b1780 Canu assembler v.1.8 using the default parameters (26). The quality analysis of the assembly was performed with Racon. The complete Sl00103 genome was uploaded to the Bacterial and Viral Bioinformatics Resource Center (BV-BRC) web server (https://www.bv-brc.org/) for gene prediction and functional annotation with the RASTtk pipeline.

### Phylogenetic tree construction

Whole-genome sequencing of Sl00103 was submitted to Geneious prime software online tool (27). The genetic distance was modeled using Jukes and Cantor, and the phylogenetic tree was constructed using the neighbor-joining method. Seventeen closely related bacterial genomes and *Bacillus subtilis* as an outgroup were included to construct the phylogenetic tree.

## RESULTS

### Isolation and preliminary screening of the bioactive isolates

We explored bacteria isolated from soil and liquid samples collected from different sites in the Afar region. A total of 301 colonies were isolated and screened for antimicrobial activity using agar plug (Fig. S2). The highest number of isolates, 63 (20.93%), originated from Sulfur Lake, followed by 55 (18.27%) from Aferera and 54 (17.94%) from Alalobad. The lowest number, 3 (0.99%), was isolated from Asahil Lake. In the preliminary screening using the agar plug diffusion method, 68 isolates (22.6%) exhibited antimicrobial activity. Among these, 49 (72.1%) showed activity against bacterial pathogens, while 19 (27.9%) exhibited activity against fungi (Table 2).

**TABLE 2:** Percentage of antagonistic activities of bioactive isolates from Afar region, Ethiopia, against human pathogens using agar plug diffusion method

Sample locations	Total isolates	Bioactive against bacteria	Bioactive against fungi	Total bioactive isolates
Sulfur lake	63 (20.93%)	16 (32.65%)	5 (26.31%)	21 (30.88%)
Asahil lake	3 (0.99%)	1 (2.04%)	1 (5.26%)	2 (2.94%)
Alalobad	54 (17.94%)	10 (20.40%)	4 (21.05%)	14 (20.58%)
Wosema	45 (14.95%)	10 (20.41%)	3 (15.79%)	13 (19.12%)
Amedela	36 (11.96%)	4 (8.16%)	3 (15.79%)	7 (10.29%)
Afrera1	45 (14.95%)	3 (6.12%)	0	3 (4.411%)
Afrera2	55 (18.27%)	5 (10.20%)	3 (15.79%)	8 (11.76%)
Total isolate	301	49	19	68

### Secondary screening

Sixteen isolates were selected for secondary screening based on their preliminary antibacterial activity. These isolates were tested against *S. aureus*, *E. faecalis*, *S. pyogenes*, *E. coli*, *P. aeruginosa*, *S. typhi*, *A. baumannii*, and *C. albicans* (Table 3). Among them, 13 (81.25%) isolates showed antimicrobial activity against *A. baumannii*, 12 (75.0%) against *S. typhi*, 11 (68.75%) against *E. faecalis*, *S. pyogenes*, *E. coli*, and *P. aeruginosa*, 10 (62.5%) against *S. aureus*, and 8 (50.0%) against *C. albicans*. Both Sl00103 and Ashl00101 showed antimicrobial activity against all pathogens, with Sl00103 showing the highest inhibition zone ranging from 13.7 ± 0.17 to 26.17 ± 0.7 mm. We selected isolate Sl00103 for further characterization and antimicrobial testing because it showed the highest antagonistic activity against all test pathogens.

**TABLE 3:** *In vitro* antimicrobial activity of bacterial isolates from Afar region, Ethiopia, against human pathogens using well diffusion method

Test isolates	Zone of inhibition (mm)																Rate n (%)		
	Sl00101	Sl00103	Sl00602	Af00101	Afl00101	Al00101	Al00102	Al00103	Al00106	Al00201	Al00202	Al00303	Ws00101	Ws00103	Am00101	Ashl00101	Resistance rate	Susceptibility rate	
*S. aureus*	14.17 ± 0.6	15.17 ± 0.4	14.5 ± 0.29	12.5 ± 0.76	-	-	-	-	12.17 ± 0.6	-	12.5 ± 0.87	13.8 ± 0.4	-	16.17 ± 0.4	16 ± 0.29	12.5 ± 0.76	6 (37.5)	10 (62.5)	
*E. faecalis*	15.17 ± 0.6	18.17 ± 0.4	15.3 ± 0.6	-	-	14.0 ± 0.29	-	13.67 ± 0.4	-	16.67 ± 0.4	14.33 ± 0.6	18.17 ± 0.4	-	14.83 ± 0.7	16.17 ± 0.6	16.33 ± 0.6	5 (31.25)	11 (68.75)	
*S. pyogens*	14.5 ± 0.29	14.17 ± 0.6	-	12.3 ± 0.89	12 ± 0.29	-	16.0 ± 0.29	-	13.3 ± 0.4	16 ± 0.29	13.17 ± 0.6	14.17 ± 0.73	13.17 ± 0.4	-	-	14 ± 0.58	5 (31.25)	11 (68.75)	
*E. coli*	-	19.8 ± 0.6	11.17 ± 0.4	-	13.0 ± 0.76	19.3 ± 1.17	18.17 ± 1.2	17.17 ± 0.4	-	11.5 ± 0.3	19.5 ± 0.9	18.3 ± 0.9	-	-	20.0 ± 0.9	17.3 ± 0.6	5 (31.25)	11 (68.75)	
*P.aeruginosa*	14.5 ± 0.29	18.3 ± 0.4	12.17 ± 0.73	11.3 ± 0.6	-	12.8 ± 0.9	-	-	-	15.17 ± 0.7	-	11.3 ± 0.4	19.3 ± 0.88	20.8 ± 0.9	19.0 ± 0.88	12.8 ± 0.73	5 (31.25)	11 (68.75)	
*S.typhi*	16.3 ± 0.4	12.67 ± 0.7	16.8 ± 0.6	13.3 ± 0.73	11.17 ± 0.6	11.3 ± 0.4	12.5 ± 0.76	-	17.17 ± 1.01	11.67 ± 0.7	-	-	16.3 ± 0.6	-	15.5 ± 0.23	16.17 ± 0.6	4 (25.0)	12 (75.0)	
*A.baumani*	13.17 ± 1.17	13.7 ± 0.17	12.3 ± 0.9	-	11.17 ± 0.7	11.17 ± 0.4	18.3 ± 0.6	11.0 ± 0.29	17.17 ± 0.0	16.5 ± 0.29	11.67 ± 0.73	-	15.17 ± 0.6	-	16.0 ± 0.86	20.7 ± 0.6	3 (18.75)	13 (81.25)	
*C. albicans*	14.5 ± 0.87	26.17 ± 0.7	^-^	12.8 ± 0.73	-	-	-	-	-	12.3 ± 0.73	-	12.3 ± 0.6	11.3 ± 0.6	14.5 ± 0.29	-	15.5 ± 0.29	8 (50.0)	8 (50.0)	

### Biochemical and MALDI-TOF MS test

Colony morphology and appearance/color on plates of the 16 bioactive isolates are summarized in Table S1; Fig. S3. Gram staining showed that 13 isolates (81.25%) were gram-positive. Biochemical tests demonstrated that all (16) isolates produced urease, 15 (93.75%) produced catalase, and 15 (93.75%) were sucrose and lactose fermenter. Enzymatic activities of the isolates indicated that 7 (44%) showed cellulase activity, 13 (81.25%) amylase activity, 3 (18.75%) gelatinase activity, and 8 (50.0%) proteases activity (Table S2; Fig. S4). The MALDI-TOF-MS-based identifications of 16 showed that 6 (37.5%) isolates were identified as *Brevibacterium linens*, 2 (12.5%) as *Brevibacillus brevis*, 2 (12.5%) as *Lysinibacillus sphaericus,* and the remaining singleton species (Table 4).

**TABLE 4:** Biochemical and enzymatic activities of bioactive isolates Afar region, Ethiopia^*a*^

S. no.	Isolate	MALDI-TOF MS	Biochemical and Enzymatic Activity																	
		Identified organism	GS	Cat	Cit	Ur	Glu	Suc	Lac	Gas	MR	VP	H_2_S	Mot	Ind	RCC	CMC	SH	GH	CH
1	Sl00101	*Brevibacterium linens*	+	+	−	+	−	+	+	−	−	+	−	−	−	+	−	+	−	+
2	Sl00103	*Bacillus pumilus*	+	+	+	−	+	+	+	−	−	+	−	+	−	rrs	+	+	−	+
3	Sl00602	*Lysinibacillus sphaericus*	+	+	+	+	+	+	−	−	−	−	−	+	−	rrs	+	+	−	−
4	Af00101	*Actinomyces naeslundii*	+	−	−	+	+	+	+	−	−	−	−	−	−	rrs	−	−	−	+
5	Af100101	*Acinetobacter lwoffii*	−	+	−	−	−	−	−	−	−	−	−	−	−	rrs	+	−	−	−
6	Al00101	*Brevibacterium linens*	+	+	−	+	−	+	+	−	−	+	−	−	−	+	−	+	+	+
7	Al00102	*Brevibacterium linens*	+	+	−	+	−	+	+	−	−	+	−	−	−	+	−	+	+	−
8	Al00103	*Sinomonas susongensis*	+	+	−	+	−	+	+	−	+	+	−	−	+	rrs	+	+	−	+
9	Al00106	*Lysinibacillus sphaericus*	+	+	+	+	+	+	−	−	−	−	−	+	−	rrs	−	+	−	+
10	Al00201	*Brevibacterium linens*	+	+	−	+	−	+	+	−	−	+	−	−	−	+	−	+	−	−
11	Al00202	*Corynebacterium thomssenii*	+	+	−	**−**	−	+	+	−	−	+	−	−	−	+	−	+	−	−
12	Al00303	*Brevibacterium linens*	+	+	−	+	−	+	+	−	−	+	−	−	−	+	−	+	−	+
13	Ws00101	*Brevibacillus brevis*	+	+	+	+	+	−	+	+	−	+	−	+	−	rrs	+	+	−	−
14	Ws00103	*Stenotrophomonas maltophilia*	−	+	+	+	−	−	−	−	−	+	−	+	−	rrs	+	−	+	−
15	Am00101	*Brevibacterium linens*	+	+	−	+	−	+	+	−	−	+	−	−	−	+	−	+	−	−
16	Ashl100101	*Brevibacillus brevis*	+	+	+	+	+	−	+	+	−	+	−	+	−	rrs	+	+	−	+

^*a*^ GS is gram staining; Cat, catalase; Cit, citrate; Ur, urease; Glu, glucose; Lac, lactose; Gas, gas productions; MR, methyl red; VP, Voges-Proskauer; H2S, hydrogen disulfide; Mot, motility; Ind, indole; RCC, rod-coccus cycle; rrs, remains rod shape; CMC, carboxymethylcellulose; SH, starch hydrolysis; GH, gelatin hydrolysis; CH, casein hydrolysis.

### Optimization of growth response for the strain Sl00103

To determine the optimal growth conditions for strain Sl00103, we evaluated temperature, pH, NaCl tolerance, and responses to various carbon and nitrogen sources. Growth peaked at 30°C, with the optimal range for bioactive compound production being 30°C to 35°C. The optimal pH for growth was 7, while pH 8 reduces growth. Strain Sl00103 showed the highest growth at 0.5% NaCl, with higher concentrations (1.0% and 2.0%) inhibiting growth. Glucose was the most effective carbon source, followed by fructose, and sucrose being the least effective. Yeast extract was the best nitrogen source, whereas NaNO3 was the least favorable (Fig. S5).

### Bioactivity of extract from strain Sl00103

The ethyl acetate extract (100 µg/mL) from Strain Sl00103 exhibited inhibition zones ranging from 19.5 ± 0.43 to 26.2 ± 0.4 mm against bacterial pathogens and 19.5 ± 0.44 mm against *C. albicans*. Likewise, the *n*-hexane extract (100 µg/mL) showed inhibition zones ranging from 17.17 ± 0.63 to 21.8 ± 1.2 mm against bacterial pathogens and 21.0 ± 1.01 mm against *C. albicans* (Table 5) and (Fig. S6).

**TABLE 5:** Antagonistic effect of ethyl acetate and *n*-hexane extracts from *Bacillus* sp. strain Sl00103 on both gram-positive and gram-negative bacteria and pathogenic fungus *C. albicans*^*a*^

Test pathogens	Extract		Positive control	
	EtAc	*n*-Hex	Cip	Amph.B
	(100 µg/mL)	(100 µg/mL)	10 µg/mL	(8 µg/mL)
*S.aureus*	21.17 ± 1.6	17.17 ± 0.63	20.50 ± 1.9	
*E. faecalis*	24.2 ± 1.3*	21.8 ± 1.2	21.5 ± 0.01*	
*S.pyogens*	26.2 ± 0.4*	20.8 ± 2.17	21.3 ± 0.8*	
*E. coli*	20.5 ± 0.62	15.0 ± 0.89	19.5 ± 1.9	
*P.aeruginosa*	20.5 ± 0.6	19.0 ± 0.57	20.0 ± 0.01	
*S.typhi*	19.5 ± 0.43	19.0 ± 1.64	21.0 ± 0.88	
*A.baumani*	19.5 ± 1.04	19.5 ± 0.86	20.5 ± 1.0	
*C. albicans*	19.5 ± 0.44	21.0 ± 1.01*		19.0 ± 1.01 *

^*a*^ M ± SEM, Mean and Standard Error of Mean, the experiment was in triplicate. Values significantly different from control if *P < 0.05 as analyzed by Student’s t-test.

### Evaluation of MIC, MBC and MFC

The ethyl acetate and n-hexane extracts exhibited MIC values ranging from 31.25 µg/mL to 62.5 µg/mL against the test pathogens. Corresponding MBC and MFC values aligned with their MIC values, confirming potent bactericidal and fungicidal properties (Table 6).

**TABLE 6:** MIC, MBC, MFC, MBC/MIC, MFC/MIC and MBIC

Solvent extract	Tested pathogens	MIC (µg/m)	MBC(µg/mL)	MFC(µg/mL)	MBC/MIC	MFC/MIC(µg/mL)	MBIC^*a*^(µg/mL)
EthAcE	*S. aureus*	31.25	125		4		31.25
	*E. faecalis*	31.25	62.5		2		31.25
	*S. pyogens*	62.5	125		2		62.5
	*E.coli*	31.25	125		4		31.25
	*P.aeruginosa*	62.5	250		4		62.5
	*S.typhi*	62.5	125		2		62.5
	*A.baumani*	62.5	125		2		62.5
	*C. albicans*	62.5	125	125		2	62.5
N-HexE	*S. aureus*	62.5	125		2		62.5
	*E. faecalis*	31.25	62.5		2		31.25
	*S. pyogens*	62.5	125		2		62.5
	*E.coli*	62.5	125		2		62.5
	*P.aeruginosa*	62.5	250		4		62.5
	*S.typhi*	62.5	125		2		62.5
	*A.baumani*	62.5	125		2		62.5
	*C. albicans*	62.5	125	125		2	62.5

^*a*^ MBIC is minimum biofilm inhibitions concentrations.

### GC-MS analysis

Ethyl acetate extract analysis revealed a diverse composition of bioactive compounds. The main compounds identified were (R, R)-Butane-2,3-diol (16.88%), 3-Isobutylhexahydropyrrolo [1,2 a] pyrazine-1,4-dione (13.23%), Cyclo (L-prolyl-L-valine) (10.88%), and Butanedioic acid, 2-hydroxy-2-methyl-, dimethyl ester, (2R) (3.74%). Other compounds include 1,3-Isobenzofurandione, Hexyl 3-methylbutanoate, Phenol, 2-methoxy-, and 5-Hydroxymethylfurfural accounted for abundance ranging from 1.26% to 0.65% ((Table 7). In the n-hexane Extract, the identified compounds include fatty acid methyl esters such as Tetradecanoic acid (13.12%), Hexadecanoic acid (9.51%), and pentanoic acid (8.12%). Also present were 1,2-benzenedicarboxylic acid, diethyl ester (2.502%) and cyclohexadecane P489 (2.501%). Aromatic carboxylic acid methyl esters, phenolic compounds, alcohols, and various alkane compounds were detected as well. The list of compounds extracted and the GC-MS chromatographic profiles for the ethyl acetate and n-hexane extracts are provided in (Tables S3 and S4; Fig. S7 and S8).

**TABLE 7:** Major compounds identified using ethyl acetate extract from **Strain Sl00103^*a*^**

Solvent for extraction	Compound name	Compound formula	RT	CAS	RA (%)	Chemical class
Ethyle acetate	(R,R)-Butane-2,3-diol	C4H10O2	5.535	513–85-9	16.88	Alcohol
	3-Isobutylhexahydropyrrolo[1,2 a] pyrazine-1,4-dione	C11H18N2O2	32.36	5654–86-4	13.23	Pyrrolopyrazine
	Cyclo(L-prolyl-L-valine)	C4H10O2	32.423	2854–40-2	10.88	Cyclopeptide
	Butanedioic acid, 2-hydroxy-2-methyl-, dimethyl ester, (2R)-	C7H12O5	12.626	81426–68-8	3.74	Dicarboxylic acid ester
	1,3-Isobenzofurandione	C8H4O3	19.465	85–44-9	1.26	Benzofuran derivative
	Hexyl 3-methylbutanoate	C11H22O2	15.919	10032–13-0	1.13	Esther
n-hexane	Tetradecanoic acid, 12-methyl-, methyl ester	C16H32O2	18.846	5129–66-8	13.126	Fatty acid methyl ester
	Hexadecanoic acid, methyl ester	C17H34O2	20.162	112–39-0	9.508	Fatty acid methyl ester
	Pentanoic acid, methyl ester	C6H12O2	4.443	624–24-8	8.119	Fatty acid methyl ester
	1,2-Benzenedicarboxylic acid, diethyl ester	C12H14O4	16.671	84–66-2	2.502	Phthalate ester
	Cyclohexadecane P489	C16H32	16.551	295–65-8	2.501	Cycloalkane
	13-Octadecenoic acid, methyl ester	C19H36O2	21.85	56554–47-3	2.460	Fatty acid methyl ester
	Methyl stearate	C19H38O2	22.073	112–61-8	2.062	Fatty acid methyl ester

^*a*^ RT, retention time; the percentage amount was calculated from the peak area. RA; Relative abundance.

### Antioxidant activities and IC50

The antioxidant activity of ethyl acetate and n-hexane extracts from strain Sl00103 was assessed by their ability to scavenge DPPH radicals. Across the concentration range tested (50 μg/mL to 550 μg/mL), both extracts showed increasing RSA values, indicating a strong potential to scavenge DPPH radicals with higher concentrations. ethyl acetate at 50 µg/ml exhibited approximately 58.76% RSA, while n-hexane showed about 38.77% RSA (Table S5). Additionally, IC50 values were computed to represent the concentration required for 50% inhibition of DPPH radical activity. Ethyl acetate exhibited an IC50 of 43.04277 µg/ml, implying a lower concentration is required for inhibition compared to n-hexane, which had an IC50 of 71.7367 µg/mL (Fig. S9).

### Whole genome sequencing, genome annotations, and assembly of strain Sl00103

**Fig 1:**
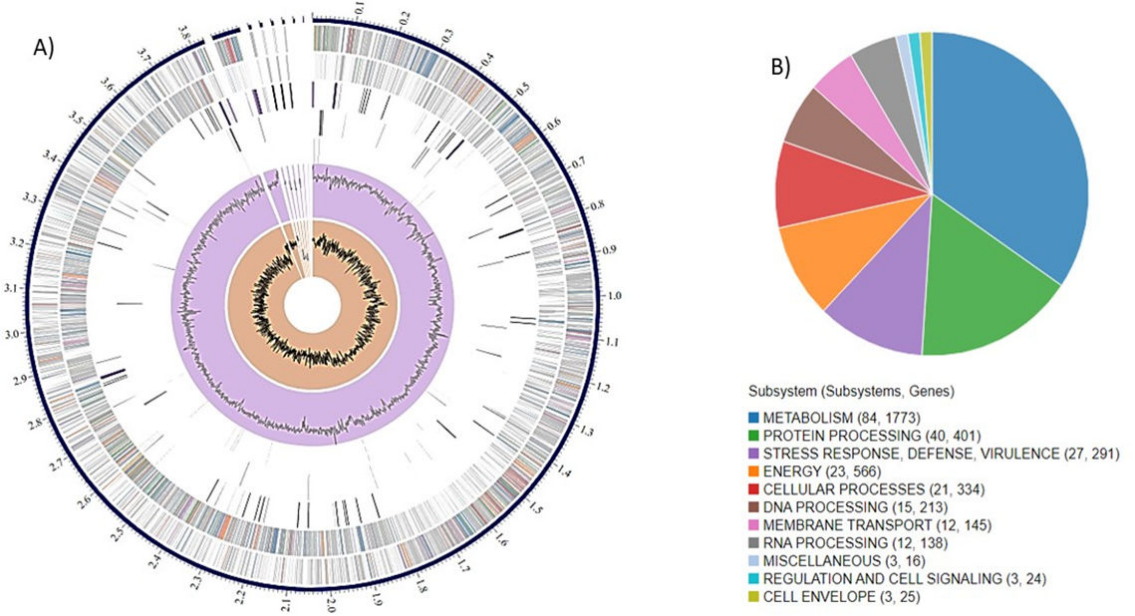
**(A**) circular graphical display of the distribution of the genome annotations of SL00103. This includes, from outer to inner rings, the contigs, CDS on the forward strand, CDS on the reverse strand, RNA genes, CDS with homology to known antimicrobial resistance genes, CDS with homology to know virulence factors, GC content and GC skew (B) pie chart showing a set of proteins with specific process or structural complex.

The genome of strain Sl00103 was sequenced and assembled into eight contigs, with a total length of 3,926,230 bp and an average G + C content of 41.03% (Fig. 1). RAST annotation predicted 77 tRNA genes, 26 rRNA genes, 4,949 hypothetical proteins, and 7,383 functionally assigned proteins.

### Phylogenetic analysis

**Fig 2:**
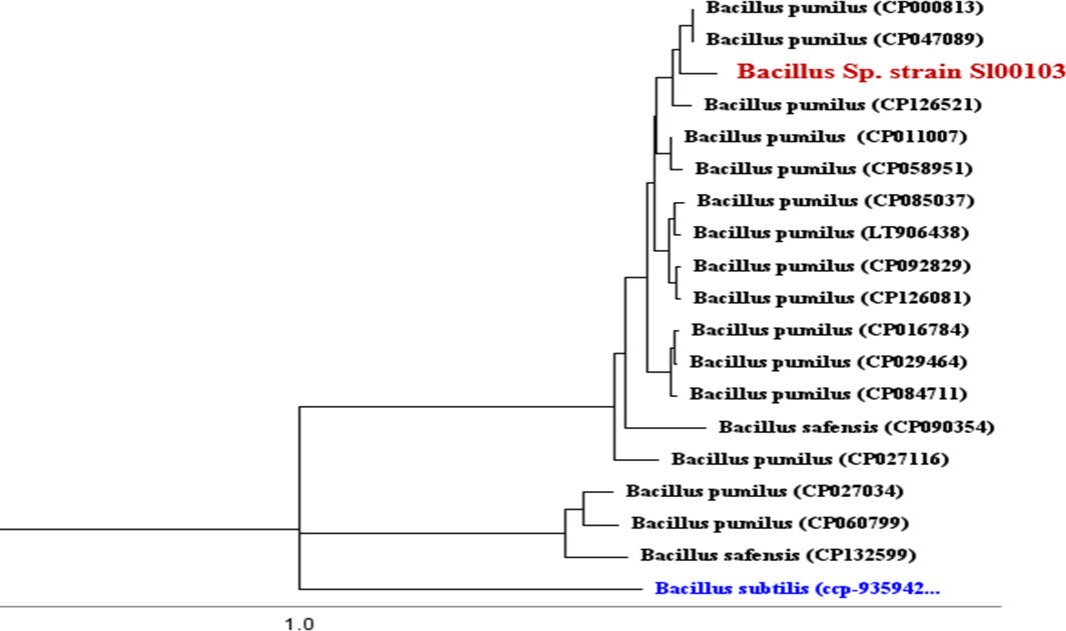
Phylogenetic placement of *Bacillus* sp. strain Sl00103 and *Bacillus subtilis*, used as the out group or reference standard.

The consensus FASTA sequence of strain Sl00103 was uploaded to NCBI Nucleotide BLAST, revealing the highest similarity (94.51%) to *Bacillus pumilus*. A phylogenetic tree was constructed using the genomes of 17 closely related bacterial species and *Bacillus subtilis* as an outgroup (Table 8) (Tables 8; Phylogenetic analysis. The analysis, conducted with Geneious Prime software, confirmed that strain Sl00103 is closely related to *Bacillus pumilus* SAFR-032, with both strains grouped in the same clade (Fig. 2). The isolates Sl00103 is, therefore, designated as *Bacillus* sp. Sl00103. Average Nucleotide Identity (ANI) analysis showed that *Bacillus* sp. Sl00103 and *Bacillus pumilus* SAFR-032 have a 94.09% ANI, below the 95%–96% species cutoff, indicating that Sl00103 is a novel strain within the *Bacillus* genus.

**TABLE 8:** List of Bacillus genomes selected for phylogenetic analyses and the overall genome-related index (OGRIs) calculation^*a*^

S.no	Strains	Accessions number	Source /Origin	Genome level	Gs (Mb)	GC%	Identity %	Reference/submitter to NCBI
1.	Bacillus Sp. Strain Sl0103	-	Desert soil	Complete genome	3.8	41.03	94.51	This study
2.	*Bacillus pumilus* strain 150 a	CP027034.1	Sediment top	Complete genome	3.7	41.5	94.91	(28)
3.	*Bacillus pumilus* strain Monterrey_S2	CP126521.1	Soil	Complete genome	3.8	41.5	94.83	(29)
4.	*Bacillus pumilus* strain B2	CP126081.1	forest soil	Complete genome	3.8	41.5	93.87	(29)
5.	*Bacillus pumilus* strain NCTC10337	LT906438.1	Not defined	Complete genome	3.9	41.5	93.86	NA
6.	*Bacillus pumilus* MS32	CP092829.1	Soil	Complete genome	3.8	41.5	93.84	NA
7.	*Bacillus pumilus* strain BIM B-171	CP085037.1	soil	Complete Genome	3.8	41.5	93.81	IMNAS, Belarus
8.	*Bacillus pumilus* SH-B9	CP011007.1	Sugar beet rhizosphere	Complete Genome	3.9	41.5	93.80	Wageningen University
9.	*Bacillus pumilus* AR03	CP084711.1	Not defined	Complete Genome	3.7	42	93.39	ITRCAAS
10.	*Bacillus pumilus* ZB201701	CP029464.1	Not defined	Complete Genome	3.6	42	93.37	BAAFC
11.	*Bacillus pumilus* PDSLzg-1	CP016784.1	il sands	Complete Genome	3.7	42	93.35	CAAS
12.	*Bacillus pumilus* 145	CP027116.1	Sediment top	Complete Genome	3.9	41	91.97	Centro de Investigation y de Estudios Avanzados del IPN - Irapuato
13.	*Bacillus pumilus* SAFR-032	CP000813.4	Not defined	Complete Genome	3.7	41.5	94.92	(28)
14.	*Bacillus pumilus* UAMX	CP058951.1	feces	Complete Genome	3.9	41.5	93.58	(30)
15.	*Bacillus pumilus* ONU 554	CP060799.1	Black sea sediments	Complete Genome	3.7	41.5	93.10	Odesa I.I. Mechnykov National University
16.	*Bacillus pumilus* MS32	CP092829.1	Soil	Complete Genome	3.8	41.5	93.84	Georg-August-University Goettingen
17.	*Bacillus safensis* PL23A	CP132599.1	Not defined	Complete Genome	3.7	41.5	89.37	Shandong First Medical University & Shandong Academy of Medical Sciences
18.	*Bacillus safensis* H31R-08	CP090354.1	Soil	Complete Genome	3.7	41.5	89.34	National Institute of Agricultural Sciences (south Korea)
19.	Bacillus subtilis subsp. subtilis str. 168	CP935942	Not defined	Complete Genome	4.2	43.5	-	British society of neuro radiologists (BSNR)

^*a*^ Gs Mb: genome sequence in mega base; IMNAS Belarus:Institute of Microbiology National Academy of Sciences of Belarus; ITRCAAS: Institute of Tobacco Research Chinese Academy of Agricultural Science; BAAFC: Beijing Academy of Agriculture and Forestry Sciences; CAAS:Chinese Academy of Agricultural Sciences, NA: not addressed

## DISCUSSION

We identified bacterial isolates from samples collected across various sites in the Afar region, characterized by extreme conditions such as active volcanic activity, high ambient temperatures (40–50°C), high salt concentrations, and hot springs (31). These environments are known for their diverse bacterial populations and potential to produce valuable secondary metabolites (32, 33). Our study isolated bacteria from these extreme conditions, aligning with previous research that highlights extreme environments as rich sources of novel bacterial species with antimicrobial potential. For instance, Batch et al. (31) recently used a metagenomic approach to identify diverse bacterial populations in the same area, underscoring these environments as promising sources for discovering new bacterial species. Similarly, Guta et al. (34) identified and characterized 252 bacterial isolates from hot springs in central Ethiopia, each demonstrating potential for at least one extracellular hydrolytic enzyme activity. This substantiates that extreme environments continue to harbor diverse bacterial isolates with potential industrial applications.

Soil hosts diverse microbial communities adapted to dynamic conditions, fostering competition among bacteria. This competition leads to the production of antimicrobial compounds, making soil bacteria ideal candidates for antibacterial screening. Accordingly, 22.6% of the identified isolates in this study showed antimicrobial effects against test pathogens. Our findings are similar to those of Tawiah et al. (35) and Amankwah et al. (36) who reported 20.85% and 20.83% bioactive bacterial isolates, respectively, but are significantly higher than the 1.14% and 3.44% bioactive isolates reported by Selvin et al. (37) and Prashanthi et al. (33). These differences may be due to variations in geographic location, sample sources, and methods used. The varying bioactivity profiles of these isolates could also be attributed to differences in the bioactive secondary metabolites they produce. Research conducted by Sharma and Thakur (38) and Elias et al*.* (39) highlighted that the diversity of natural habitats, where isolates originate from, influences their capacity for secondary metabolite production. The high rate of bioactive bacteria in our study indicates the potential of sampled environments for discovering bioactive compounds. Moreover, the isolated bioactive bacteria demonstrated the production of enzymes crucial for biotechnological applications, such as cellulose, starch, gelatin, and casein. This study aligns with research by Manni and Filali Maltouf (40) and Valenzuela et al. (41), which reported that thermo-tolerant bacteria produce various hydrolytic enzymes, suggesting further biotechnological potential.

In addition to preliminary screening, we demonstrated the antibacterial potential of 16 selected bacterial isolates in secondary screening, showing antimicrobial activity against at least three test pathogens. These findings are consistent with previous research highlighting the potent antimicrobial effects of these species against human pathogens and the fungus *C. albicans*. For instance, significant antimicrobial activity has been reported for *Bacillus* sp. (42, 43), *Brevibacterium linens* (44), *Brevibacillus brevis* (45), *Lysinibacillus* sp. (46, 47), *Stenotrophomonas maltophilia* (48), *Actinomyces naeslundii,* and *Acinetobacter lwoffii* [reviewed in reference (49)]. These findings underscored the potential of these microbes for producing secondary metabolites with antimicrobial activity.

Given its broad antimicrobial activities against various test pathogens and the strength of its inhibition zone, strain Sl00103, identified as *Bacillus pumilus* Sl00103, was further characterized through whole-genome sequencing (WGS). The analysis revealed that it is very closely related to *Bacillus pumilus* SAFR-032, with both strains grouped in the same clade while the ANI analysis suggested that it is novel strain. Several studies have demonstrated that this bacterial species is known for its potent antimicrobial activities (50–52). The MIC of the extract against the tested pathogens indicated inhibitory effects. The antioxidant activity of SL00103 extracts was assessed through DPPH assays, revealing increasing scavenging capacities and suggesting their potential as free radical inhibitors (53).

GC-MS analysis of the strain Sl00103 extract revealed that (R,R)-Butane-2,3-diol was the predominant compound in the ethyl acetate extract, comprising 16.88% of the secondary metabolites. Also known as 2,3-butylene glycol, 2,3-Butanediol (2,3-BDO) is a metabolic product excreted by various microorganisms, including *Bacillus subtilis*, *B. amyloliquefaciens*, *Klebsiella oxytoca*, *K. pneumoniae*, *Enterobacter cloacae*, and *Serratia marcescens* (54). It serves as a valuable precursor for the synthesis of chemicals and exhibits antimicrobial activity (55). Closely following in abundance was 3-isobutylhexahydropyrrolo[1,2a] pyrazine-1,4-dione, a pyrrolopyrazine compound representing 13.23% of the secondary metabolite profile. This compound belongs to the diketopiperazine (DKP) class, which is notable in medicinal chemistry for its stable six-membered ring structure and potential as a pharmacophore (56). This compound also exhibited inhibitory effects against pathogenic bacteria (57); exhibited quorum-sensing inhibition (58); inhibits biofilm formation and pyocyanin production in *P. aeruginosa* PAO1 (59); Similarly, the ethyl acetate extract of *Bacillus pumilus* S8-07 inhibits biofilm formation in *P. aeruginosa* PAO1 (60). Another significant metabolite identified was Cyclo (L-prolyl-L-valine), a Cyclopeptide. As detailed by Sánchez-Tafolla et al. (61), cyclopeptides exhibit a diverse range of biological functions, including acting as siderophores, cellular signaling molecules, antimicrobial agents, and cytotoxic agents against specific cancer cell lines.

GC-MS analysis of strain Sl00103 using *n*-hexane revealed a diverse array of compounds. The dominant compound was tetradecanoic acid, 12-methyl-, methyl ester, which constituted 13.13% of the extract. This compound exhibits activity against *Erwinia amylovora* and *Xanthomonas ampelinus* (62) and shows antifungal properties (63). Another significant compound was hexadecanoic acid, methyl ester (C₁₇H₃₄O₂), representing 9.51% of the extract. This fatty acid methyl ester has various applications, including antibacterial activity (64), as well as anti-inflammatory, nematicide, pesticide, lubricant, anti-androgenic, flavoring, hemolytic, 5-alpha reductase inhibition, antioxidant, and hypocholesterolemic effects (65).

### Conclusion

This study isolated several promising bacterial strains from the Afar region in Ethiopia, with strain SL00103 (*Bacillus* sp. Sl00103) demonstrating notable antimicrobial and antioxidant activities against various pathogens. GC-MS analysis revealed several antimicrobial compounds, suggesting that strain SL00103 is a valuable source of secondary metabolites with significant potential for pharmaceutical applications and justifying further studies.

## Data Availability

The genomes of the *Bacillus sp*. Sl00103 was submitted to the NCBI GenBank and was assigned the accession number SAMN43404352.

## SUPPLEMENTAL MATERIAL

Supplemental material spectrum.01810-24-s0001.pdf Contains all the necessary information and data generated during the execution of the research project.

ASM does not own the copyrights to Supplemental Material that may be linked to, or accessed through, an article. The authors have granted ASM a non-exclusive, world-wide license to publish the Supplemental Material files. Please contact the corresponding author directly for reuse.
