# Supplementary material for: Diversity of culturable bacterial isolates and their potential as antimicrobial against human pathogens from Afar region, Ethiopia
Source: Microbiol Spectr. 2024 Oct 4;12(11):e01810-24. doi: 10.1128/spectrum.01810-24 (PMC11537106; doi:10.1128/spectrum.01810-24)
Supplement: Supplemental material — Contains all the necessary information and data generated during the execution of the research project. [file spectrum.01810-24-s0001.pdf]

Diversity of Culturable Bacterial Isolates and Their Potential as Antimicrobial Against Human Pathogens from Afar Region, Ethiopia

Sisay Demisie[1], Dong-Chan Oh[2], Dawit Wolday[3], Tobias F. Rinke de Wit[4], Adugna Abera[5], Geremew Tassew[5], Abebe Mekuria Shenkutie[6], Ketema Tafess[1&7]*

[1]Department of Applied Biology, School of Applied Natural Science, Adama Science and Technology University, P.O. Box 1888, Adama, Ethiopia

[2]Natural Products Research Institute, College of Pharmacy, Seoul National University, Seoul 08826, Republic of Korea

[3]Dept of Biochemistry & Biomedical Sciences, McMaster University, Canada

[4]Amsterdam Institute for Global Health and Development (AIGHD), Department of Global Health, Amsterdam University Medical Centre, University of Amsterdam, Amsterdam, the Netherlands

[5]Ethiopian Public Health Institute, Addis Ababa, Ethiopia

[6]Department of Health Technology and Informatics, The Hong Kong Polytechnic University, Hong Kong SAR, China

[7]Institute of Pharmaceutical Sciences, Adama Science and Technology University, P.O. Box 1888, Adama, Ethiopia

Contents

Supplementary Figure 1 3
Supplementary Figure 2 3
Supplementary Table 1 4
Supplementary Figure 3 5
Supplementary Table 2 6
Supplementary Figure 4 7
Supplementary Figure 5 8
Supplementary Figure 6 9
Supplementary Table 3 10
Supplementary Figure 7 11
Supplemantary Table 4 12
Supplementary Figure 8 13
Supplementary Table 5 14
Supplementary Figure 9 15

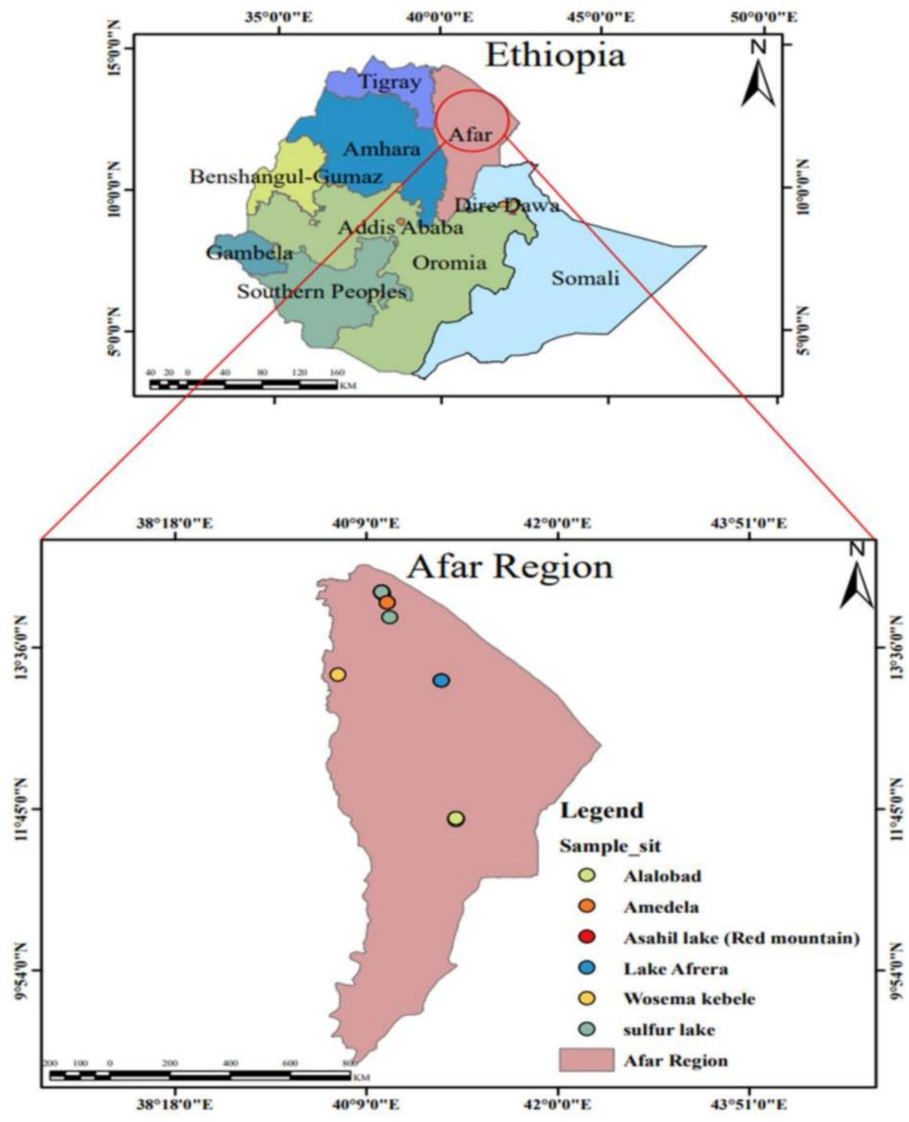
Ethiopia
Tigray
Afar
Amhara
Benshangul-Gumaz
Dire Dawa
Addis Ababa
Gambela
Oromia
Somali
Southern Peoples
Afar Region
Legend
Sample_sit
Alalobad
Amedela
Asahil lake (Red mountain)
Lake Afrera
Wosema kebele
sulfur lake
Afar Region

Supplementary Figure 1: Map of the sample collection sites in the Afar region. The geographical coordinates were obtained using the MAPS.ME application; January 2023

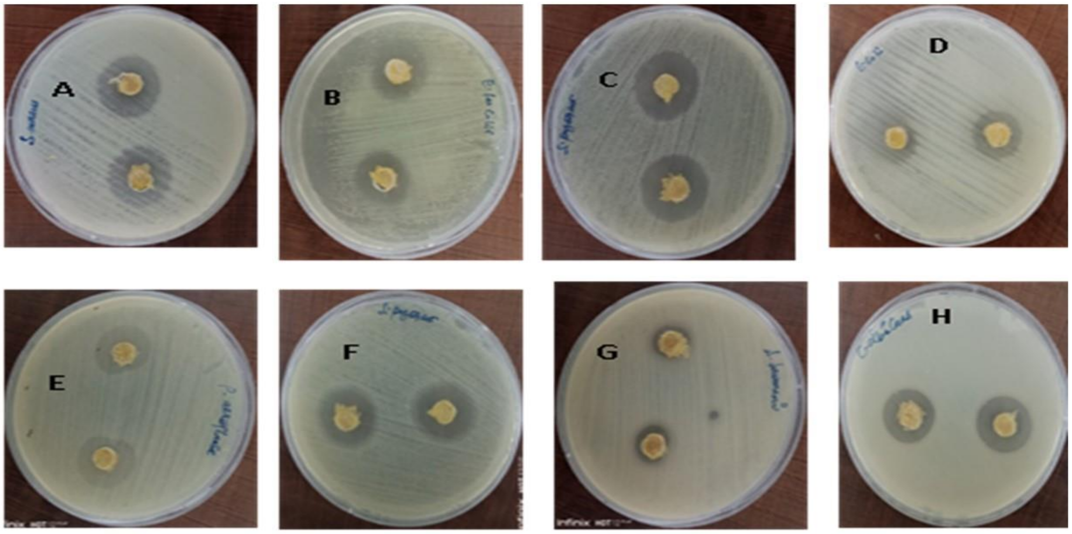

Supplementary Figure 2*:* Representative figure showing agar plug based preliminary screening of bioactive isolates (Sl00103). A, B, C, D, E, F, G and H represent isolates screened by agar plug diffusion method, demonstrating clear zones of inhibition against *S. aureus, E. faecalis, S. pyogens, E. coli, P.aeruginosa, S. typhi, A.baumani* and *C. albicans,* respectively

Supplementary Table 1. Morphological characterizations of the bioactive isolates from Dallol Depression and other areas in Afar region, Ethiopia on culture plates

	**Morphological characterizations using microscopic and appearance on culture plates**						
**S.No**	***Isolate code***	***Form/shape***	***Size***	***Elevation***	***Margin***	***Surface***	***Color***
1	Sl00101	Rods	Large	Flat	Smooth	Smooth	White
2	Sl00103	Rods	Large	Flat	Smooth	Smooth	White
3	Sl00602	Circular	Large	Raised	Entire	Smooth	White
4	Afl00101	Rods	Large	Raised	Undulate	Glistening	Light brown
5	Al00101	Rods	Medium	Flat	Entire	Smooth	White
6	Al00102	Circular	Medium	Flat	Entire	Smooth	Milky white
7	Al00103	Rods	Medium	Flat	Entire	Smooth	Light yellow
8	Al00106	Rods	Medium	Flat	Undulate	Smooth	White
9	Al00201	Irregular	Medium	Flat	Smooth	Smooth	Brown
10	Af00101	Filamentous	Large	Flat	Undulate	Rough	yellow
11	Al00303	Circular	Large	Umbonate	Filamentous	Smooth	White
12	Al00202	Circular	Large	Raised	Entire	Smooth	Light brown
13	Am00101	Rods	Large	Flat	Entire	Smooth	white
14	Ws00101	Circular	Medium	Umbonate	Undulate	Wrinkled	White
15	Ws00103	Rods	Small	Umbonate	Entire	Wrinkled	Milky White
16	Ashl100101	Pleomorphic	Medium	Dual raised & umbonate	Undulate	Wrinkled	White creamy

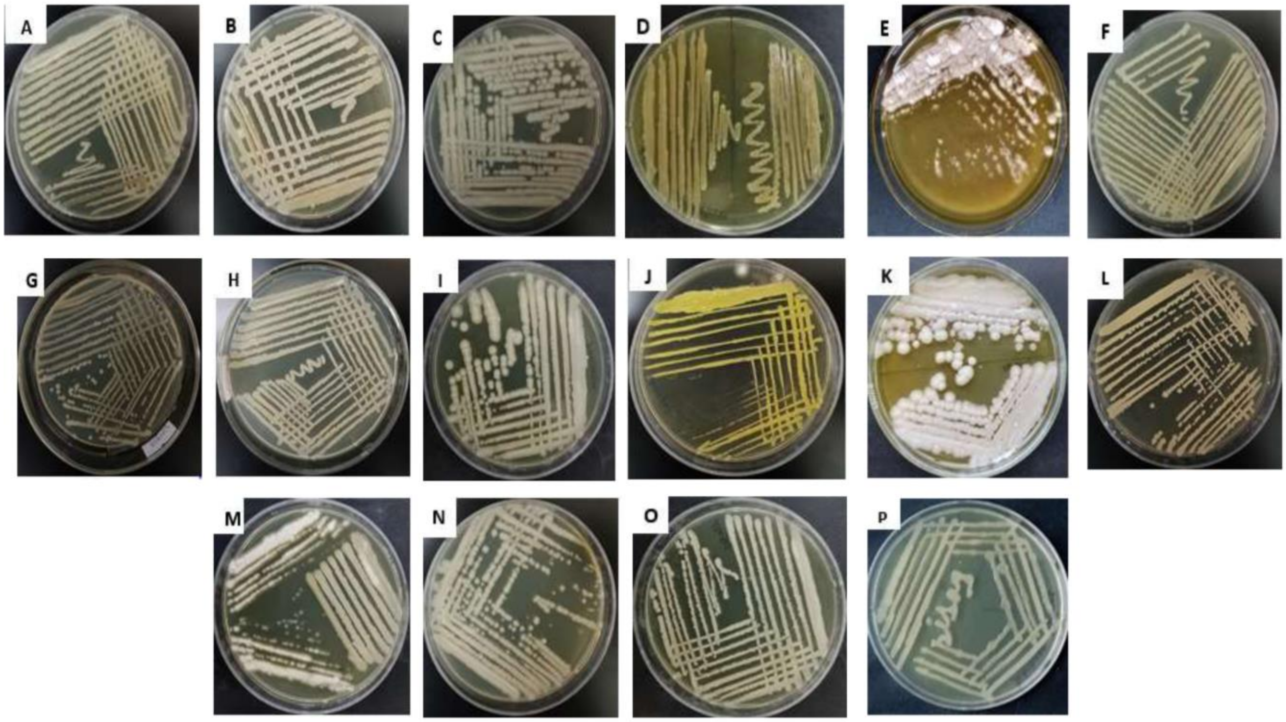

Supplementary Figure 3: Representative plates of 16 bioactive isolates obtained from Sulfur Lake (A-C): Sl00101, Sl00103, Sl00602; Afrera (D-E): Af00101, Afl00101; Alalobad, (F-L): Al00101, Al00102, Al00103, Al00106, Al00201, Al00303, Al00202; Amedela (M): Am00101; Wasema (N-O): Ws00101, Ws00103; Asahil lake (P): Ashl100101

(N): Ashl100101;

Supplementary Table 2. Clearing index for the determination of hydrolytic enzymes activities in isolated bacteria

**S.No**	**Isolate code**	**CMC**			**SH**			**GH**			**CH**		
		**C/size**	**C/zone (Mean)**	**CI (M ± SEM)**	**C/size**	**C/zone**	**CI (M ± SEM)**	**C/size**	**C/zone**	**CI (M ± SEM)**	**C/size**	**C/zone**	**CI (M ± SEM)**
1.	Sl00103	3	13	4.33±0.02	5	8	1.6±1.2	NA	NA	No	5	15	3±0.104
2.	Sl00101	NA	NA	NA	2	3	1.5±1.2	3	5	1.66±1.2	5	13	2.6±0.04
3.	Sl00602	3	12	4.0±1.021	5	11	2.5±1.2	NA	NA	No	NA	NA	NA
4.	Af00101	3	10	3.33±0.02	NA	NA	NA	NA	NA	No	NA	NA	NA
5.	Afl00101	NA	NA	NA	3	5	1.66±1.2	NA	NA	No	NA	NA	NA
6.	Al00101	NA	NA	NA	3	5	1.66±1.2	3	15	5±1.22	5	13	2.6±1.20
7.	Al00102	NA	NA	NA	NA	NA	NA	2	3	1.5±1.45	5	15	3±0.104
8.	Al00103	NA	NA	NA	3	5	1.66±1.2	2	3	1.5±1.45	5	15	3±0.104
9.	Al00106	NA	NA	NA	NA	NA	NA	NA	NA	NA	NA	NA	NA
10.	Al00201	NA	NA	NA	NA	NA	NA	NA	NA	NA	NA	NA	NA
11.	Al00202	NA	NA	NA	NA	NA	NA	NA	NA	NA	NA	NA	NA
12.	Al00303	NA	NA	NA	NA	NA	NA	NA	NA	NA	5	13	2.6±0.14
13.	Ws00101	3	14	4.66±1.3	NA	NA	NA	NA	NA	NA	NA	NA	NA
14.	Ws00103	3	16	5.33±0.5	NA	NA	NA	NA	NA	NA	NA	NA	NA
15.	Am00101	NA	NA	NA	NA	NA	NA	3	12	4.0±.61	NA	NA	NA
16.	Ashl00101	3	15	5.00±0.5	NA	NA	NA	5	8	1.6±1.2	NA	NA	NA

Note: CMC; Carboxymethylcellulose, SH; Starch hydrolysis, GH; Gelatin hydrolysis, CH; Casein hydrolysis

NB: Calculate the clearing index: The clearing index was calculated as the ratio of the diameter of the clearing zone to the diameter of the bacterial colony. This ratio provides a quantitative measure of the extent of hydrolysis relative to the size of the colony. Clearing index=diameter of clearing zone/diameter of bacterial colony.

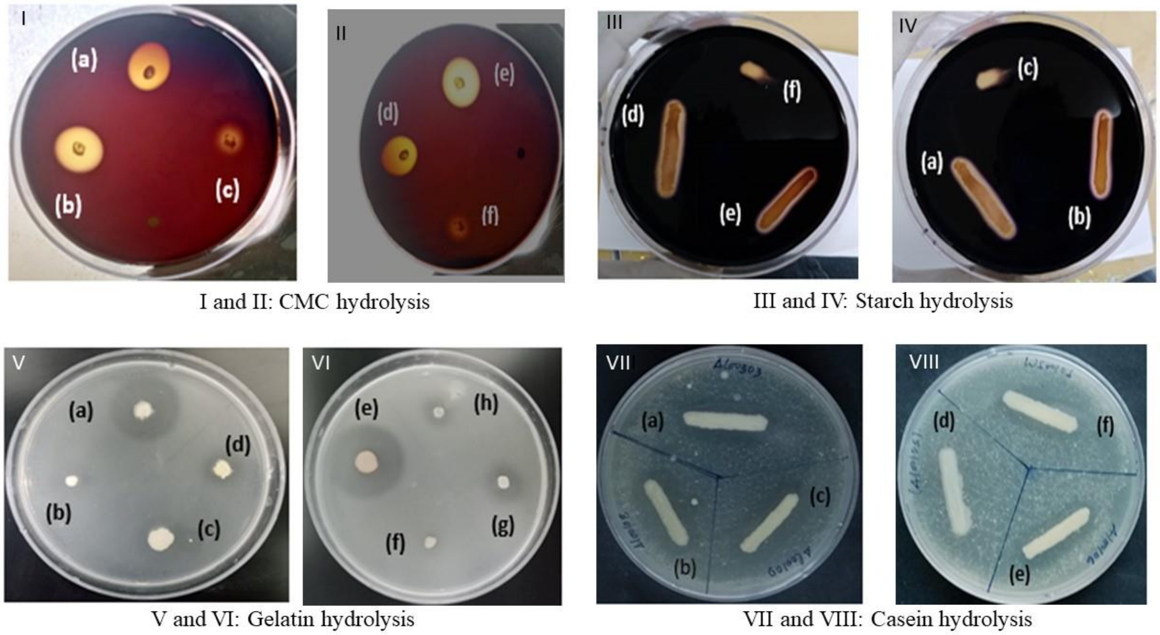

Supplementary Figure 4:

- Panels I and II: Display bioactive isolates with cellulase-degrading activity. The isolates are: Sl00103 (a) and Sl00602 (b) from Sulfur Lake (brine salt), Af00101 (c) from Afrera Lake (brine lake), Ws00101 (d) and Ws00103 (e) from Wosema Kebele, Ashl00101 (f) from Asahil Lake (brine lake).
- Panel IV: Shows bioactive isolates with starch-degrading activity. The isolates are: Sl00602 (a), Sl00103 (b), and Sl00101 (c) from Sulfur Lake (brine salt), Al00101 (d) and Al00103 (e) from Alalobad, Afl00101 (f) from Afrera Lake (brine lake).
- Panels V and VI: Illustrate bioactive isolates with gelatin-degrading activity. The isolates are: Al00101 (a) and Al00102 (c) from Alalobad, S00101 (d) and Sl00103 (g) from Sulfur Lake (brine salt), Am00101 (e) and Ashl00101 (h) from Asahil Lake (brine lake).
- Panels VII and VIII: Show bioactive isolates with casein-degrading activity. The isolates are: Sl00101 (a) and Sl00103 (b) from Sulfur Lake (brine salt), A00101 (c), Al00102 (d), A00103 (e), and A00303 (f) from Alalobad.

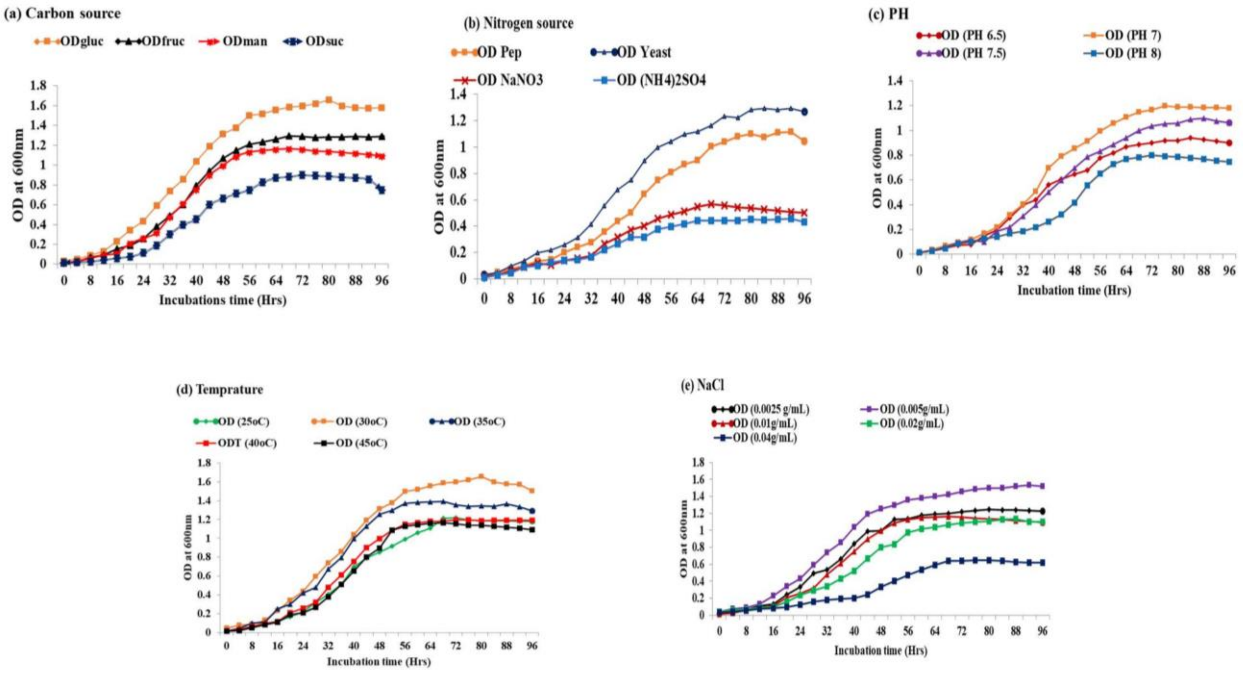

Supplementary Figure 5: 9a, 9b, 9c, 9d and 9e are represented comparison of OD at 600nm in relation to different carbon, nitrogen's, P^H level, temperature, and different concentrations of NaCl tolerance for Sl00103.

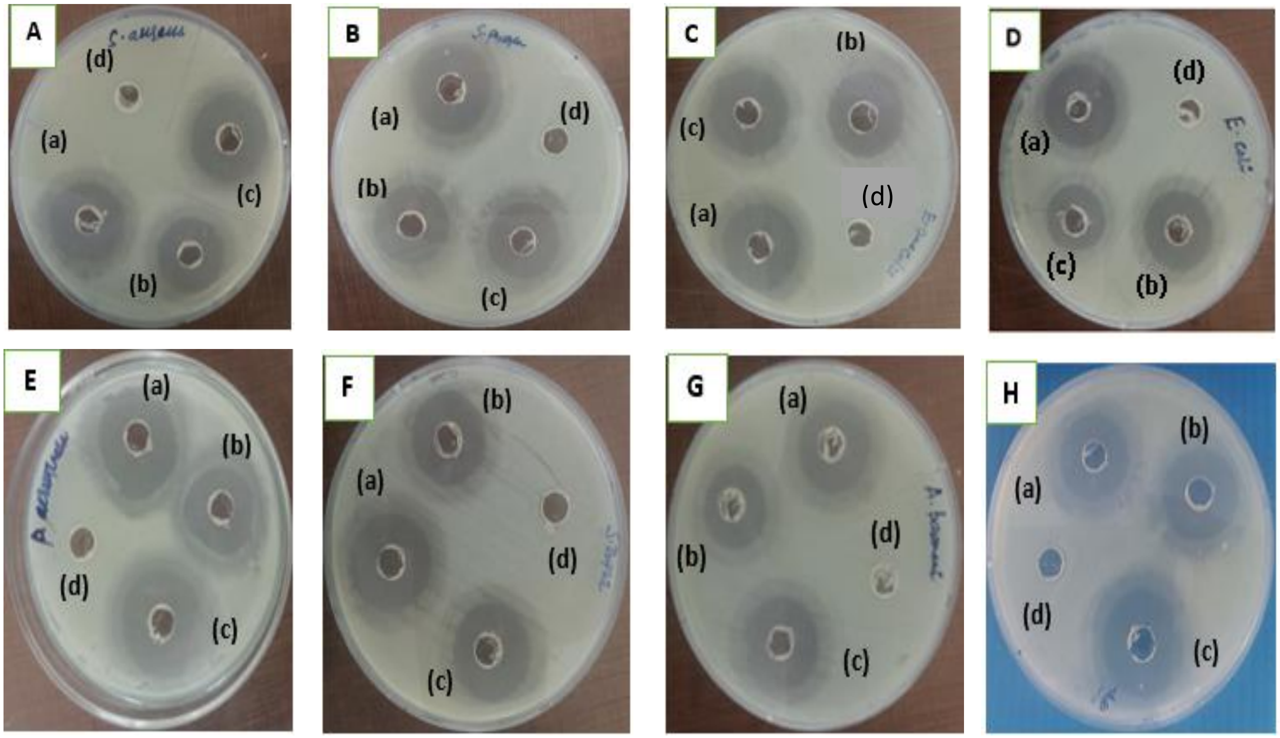

Supplementary Figure 6: Antagonistic effect of EthAcE and n-Hex extract of Sl00103 against the reference pathogens A) *S. aureus*, B) *S. pyogens* C) *E. faecalis*, D) *E. coli*, E) *P. aeruginosa*, F) *S. typhi*, G) *A. baumani* and H) *C. albicans*. In additions, the lowercase represented a) ethyl acetate b) n_HexE c) +ve control Ciprofloxacin (10µg/mL) for antibacterial and Amphotericin B liposome (8µg/mL) antifungal d) –ve control (10%DMSO).

Supplementary Table 3. Major compounds identified using ethyl acetate extract from Sl00103, bioactive isolates from Dallol Depression in Afar region, Ethiopia

Sr N	Compound name	Compound formula	RT	CAS	RA (%)	Chemical class
1	(R,R)-Butane-2,3-diol	C4H10O2	5.535	513-85-9	16.88	Alcohol
2	3-Isobutylhexahydropyrrolo[1,2-a]pyrazine-1,4-dione	C11H18N2O2	32.36	5654-86-4	13.23	Pyrrolopyrazine
3	Cyclo(L-prolyl-L-valine)	C4H10O2	32.423	2854-40-2	10.88	Cyclopeptide
4	Butanedioic acid, 2-hydroxy-2-methyl-, dimethyl ester, (2R)-	C7H12O5	12.626	81426-68-8	3.74	Dicarboxylic acid ester
5	1,3-Isobenzofurandione	C8H4O3	19.465	85-44-9	1.26	Benzofuran derivative
6	Hexyl 3-methylbutanoate	C11H22O2	15.919	10032-13-0	1.13	Esther
7	Phenol, 2-methoxy-	C7H8O2	13.711	90-05-1	0.67	Phenol derivatives
8	5-Hydroxymethylfurfural	C6H6O3	17.496	67-47-0	0.65	Furan derivative
9	1-Hexanol, 2-ethyl-	C8H18O	11.881	104-76-7	0.61	Alcohol
10	Methyl phenyl acetate	C9H10O2	16.216	101-41-7	0.59	Esther
11	Hexadecanoic acid, methyl ester	C17H34O2	32.088	112-39-0	0.44	Fatty acid ester
12	2H-Pyran, 2-(bicyclo [2.2.1] hept-2-yloxy) tetrahydro-, (1. alpha. 2. beta., 4.alpha.)-	C12H20O2	13.591	122685-25-0	0.42	sesquiterpenoids
13	Phenyl 4 [bis(ethoxycarbonyl)but-3-ynyl]-2,3,4-trideoxy-.alpha.,L-glcero-pent-2-enopyranoside	C21H26O6	34.486	997778-64-4	0.24	Phenylpropanoic acid ester
14	Ethanone, 1-(9H-pyrido[3,4-b]indol-1-yl)-	C13H10N2O	33.401	50892-83-6	0.23	Ketone
15	2,5-Cyclohexadiene-1,4-dione, 2,5-dihydroxy-3,6-dimethyl-	C8H8O4	18.84	2654-72-0	0.17	Quinone derivative

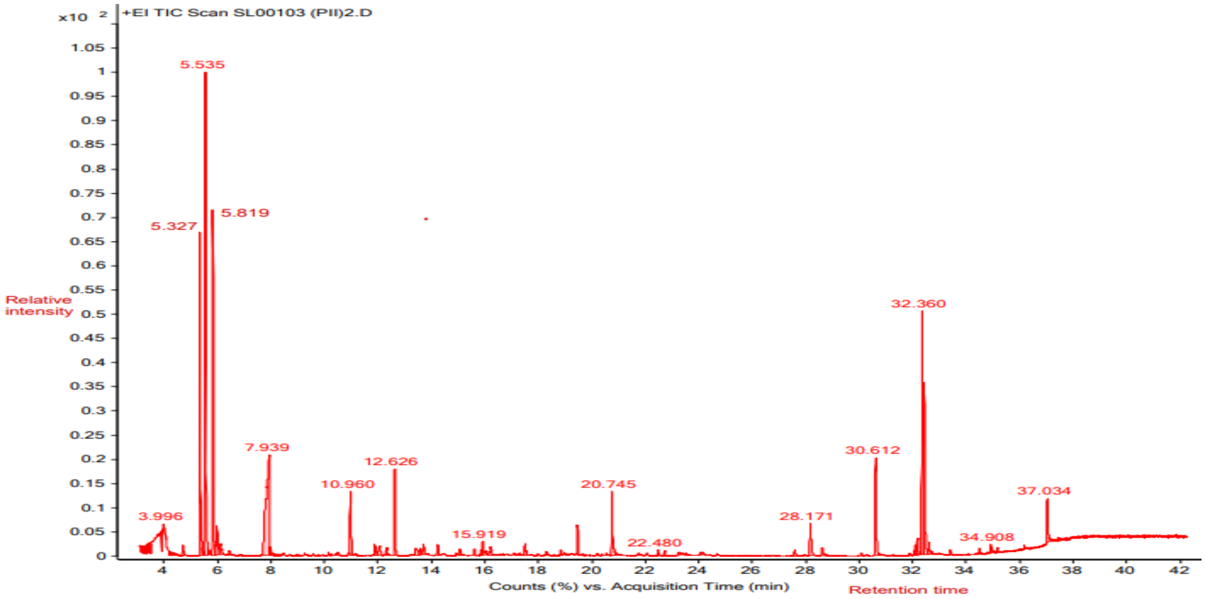

Supplementary Figure 7: GC-MS chromatographic profile of EtAc extracts from Sl00103. The peaks indicate the major compounds, and the numbers above the peaks depict the retention time. The *y*-axis is for "intensity".

Supplemantary Table 4. Major compounds identified using n-hexane extract from Sl00103, bioactive isolates from Dallol Depression Afar region, Ethiopia

SN	Compound name	Compound formula	RT	CAS	RA %	Chemical class
1	Tetradecanoic acid, 12-methyl-, methyl ester	$C_{16}H_{32}O_2$	18.846	5129-66-8	13.126	Fatty acid methyl ester
2	Hexadecanoic acid, methyl ester	$C_{17}H_{34}O_2$	20.162	112-39-0	9.508	Fatty acid methyl ester
3	Pentanoic acid, methyl ester	$C_6H_{12}O_2$	4.443	624-24-8	8.119	Fatty acid methyl ester
4	1,2-Benzenedicarboxylic acid, diethyl ester	$C_{12}H_{14}O_4$	16.671	84-66-2	2.502	Phthalate ester
5	Cyclohexadecane P489	$C_{16}H_{32}$	16.551	295-65-8	2.501	Cycloalkane
6	13-Octadecenoic acid, methyl ester	$C_{19}H_{36}O_2$	21.85	56554-47-3	2.460	Fatty acid methyl ester
7	Methyl stearate	$C_{19}H_{38}O_2$	22.073	112-61-8	2.062	Fatty acid methyl ester
8	1-Dodecanol	$C_{12}H_{26}O$	14.062	112-53-8	1.55	Alcohol
9	Benzenepropanoic acid, methyl ester	$C_{10}H_{12}O_2$	12.552	103-25-3	1.51	Aromatic carboxylic acid methyl ester
10	Phenol, 2,4-bis(1,1-dimethylethyl)-	$C_{14}H_{22}O$	15.619	96-76-4	1.32	Phenolic compound
11	Cyclohexadecane P489	$C_{16}H_{32}$	22.662	295-65-8	1.035	Alkane
12	Tetradecane P396	$C_{14}H_{30}$	14.159	629-59-4	0.866	Fatty alcohol
13	Stearyl alcohol P721	$C_{18}H_{38}O$	24.362	112-92-5	0.457	Alkane
14	Pentadecane	$C_{15}H_{32}$	15.43	629-62-9	0.436	Alkane
15	Eicosane	$C_{20}H_{42}$	24.402	112-95-8	0.391	Alkane

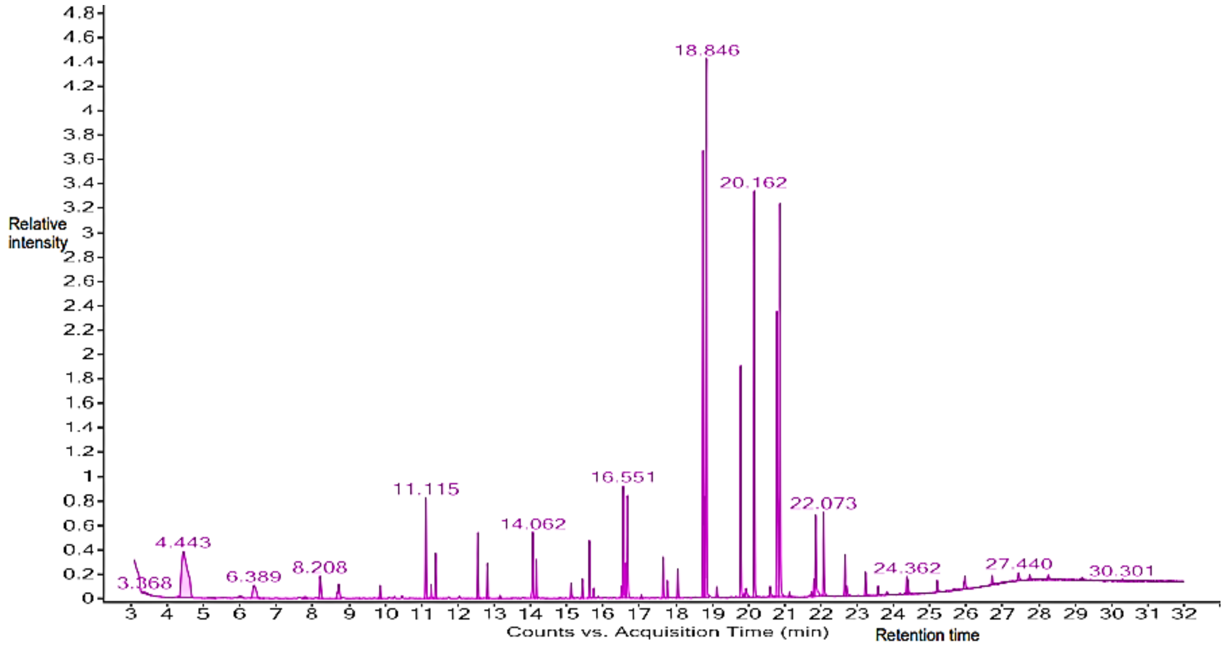

Supplementary Figure 8: GC-MS chromatographic profiles of n-hexane extract from Sl00103. The peaks indicate the major compounds, and the numbers above the peaks depict the retention time. The *y*-axis is for “intensity”.

Supplementary Table 5: Antioxidant activity of ethyl acetate and n- hexane extract of secondary metabolite from the strains Sl00103 isolates with DPPH.

**Concentrations (µg/mL)**	**EthAcE (%RSA)**	**n-hex (%RSA)**	**Ascorbic acid %RSA**
50	58.76	38.77	72.53
150	63.59	47.90	80.65
250	72.54	57.75	87.31
350	77.09	66.97	91.69
450	84.39	71.53	94.79
550	89.51	80.01	98.35

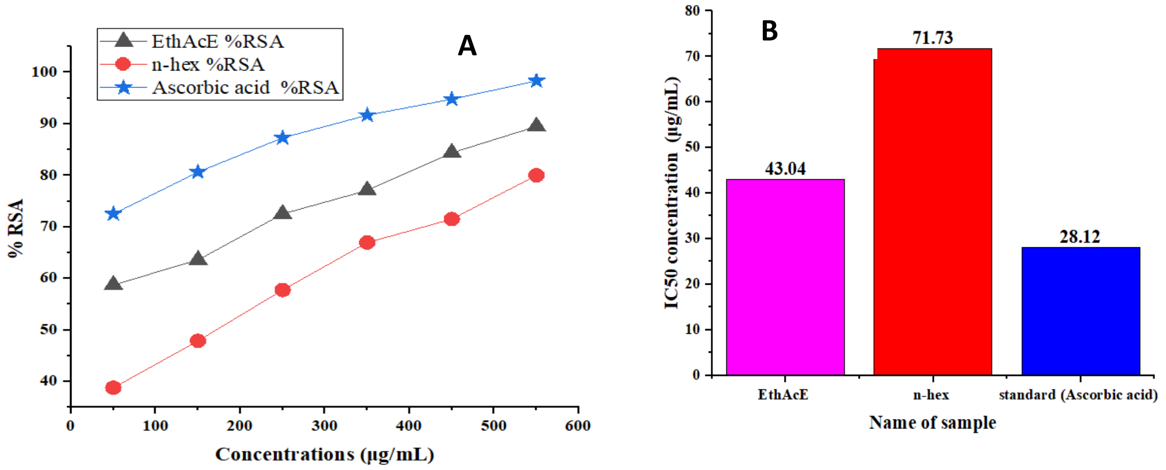

Supplementary Figure 9: 6A: Determination %RSA of EthAcE, n-hex and Standard (Ascorbic acid) for Sl00103; 6B: IC50 of EthAcE, n-hex and Standard (Ascorbic acid) for Sl00103. All experiments were performed in triplicate. Data are expressed as mean ± SD ($n = 3$, $p < 0.05$) for all tested dosages. Standard, ascorbic acid. For EthAcE and n-hexane extract, the concentration is in μg/mL.
